# Quantum many-body simulations on digital quantum computers: State-of-the-art and future challenges

**DOI:** 10.1038/s41467-024-46402-9

**Published:** 2024-03-08

**Authors:** Benedikt Fauseweh

**Affiliations:** 1 Institute for Software Technology, German Aerospace Center (DLR), Linder Höhe, 51147 Cologne, Germany; 2 Department of Physics, TU Dortmund University, Otto-Hahn-Str. 4, 44227 Dortmund, Germany

**Keywords:** Quantum simulation, Quantum information, Topological matter

## Abstract

Simulating quantum many-body systems is a key application for emerging quantum processors. While analog quantum simulation has already demonstrated quantum advantage, its digital counterpart has recently become the focus of intense research interest due to the availability of devices that aim to realize general-purpose quantum computers. In this perspective, we give a selective overview of the currently pursued approaches, review the advances in digital quantum simulation by comparing non-variational with variational approaches and identify hardware and algorithmic challenges. Based on this review, the question arises: What are the most promising problems that can be tackled with digital quantum simulation? We argue that problems of a qualitative nature are much more suitable for near-term devices then approaches aiming purely for a quantitative accuracy improvement.

## Abstract (web summary)

Digital quantum simulations of quantum many-body systems have emerged as one of the most promising applications of near-term quantum computing. This Perspective article provides an overview and an outlook on future developments in this field.

## Introduction

One of the main problem classes a quantum computer can tackle is the simulation of quantum many-body systems. Creating and controlling novel states of quantum matter is a driver in solid-state physics with many potential applications. Understanding the complex phase diagram of the cuprate superconductors^1^, the underlying mechanism of many-body localization^2^ and the behavior of quantum systems out of equilibrium^3,4^ are key questions that are difficult to answer even with the best supercomputers currently available.

The reason that quantum simulation is a computationally hard problem is due to the tensor product structure of the Hilbert space for combined quantum systems, i.e., the many-body Hilbert space forms as a tensor product of the individual Hilbert spaces for each degree of freedom^5^. For example, consider a quantum system with *N* two-level systems (qubits). The dimension of the Hilbert space for each qubit is 2, and the dimension of the Hilbert space for the entire system is determined by the tensor product of the individual Hilbert spaces, which is 2^*N*^. Thus, as the size of the quantum system increases, the dimension of the Hilbert space grows exponentially with *N*. Modern classical algorithms, such as tensor networks^6^ and neural network quantum states^7^, try to limit the impact of the exponential scaling by using a low-rank representation of quantum states. However, this approach is not always applicable, as many physical states do not possess such a representation, especially for highly entangled quantum matter in two and higher dimensions and in systems out of equilibrium.

One way to tackle this issue is to find a quantum platform that acts as a surrogate for the system one wants to investigate, i.e., analog quantum simulation. Control parameters are used to tune the behavior of the physical quantum platform to match that of the system and by measuring the state on the platform, it is possible to learn about the dynamics of the system being studied. Analog quantum simulation has been a very successful method in the recent decade in order to simulate quantum systems that are out of reach for classical approaches, with quantum advantage demonstrated using trapped ions and ultra cold atom experiments^8–18^. Here quantum advantage denotes to the ability of quantum devices to efficiently model and predict quantum many-body systems, surpassing the computational limits of classical computers.

In contrast, digital quantum simulation (DQS) uses a gate-based quantum computer to simulate the quantum system^19^. In its original formulation it involves discretizing the time evolution of the system and breaking it up into a series of small time steps, which can be implemented using quantum gates^20^. This definition has been broadened in recent years to describe any kind of gate-based quantum simulation of quantum systems. DQS has the potential advantage over analog quantum simulation that it allows for universal simulation of many-body dynamics, particularly for systems that do not ’fit’ onto analog quantum simulators^21^. For example, it might be impossible to simulate certain many-body interactions as they are physically not realizable in hardware, but its digital decomposition can very well be implement on a gate-based platform.

### Quantum computing platforms

Since Feynman’s original proposal of a quantum computer^19^ various technologies have emerged as potential platforms for gate based quantum computing. Here we give a short overview of some of the pursued approaches that have already been used for analog and digital quantum simulation.

Laser cooled neutral atoms have been very successful for analog quantum simulation. This technology has recently also reached a level which allows for digital quantum computation^22^. In this case qubits are realized as electronic states of the atoms, which are entangled via long-range Rydberg states. Recent developments in cold atom quantum computing have led to the creation of quantum processors with dynamic, non-local connectivity, enabling coherent transportation of entangled qubits across two dimensions and the ability to individually address single atoms, capable of executing quantum algorithms like quantum phase estimation (QPE) and producing entangled graph states^23,24^.

Another promising platform is trapped ions^25–27^. Trapped ions were one of the first platforms that were proposed as practical quantum computers^28^, with the first quantum gate implemented in 1995^29^. Trapped ions have a long coherence time, as the qubits are formed by internal electronic states which are well isolated. The ions are confined by dynamic electromagnetic fields (Pauli traps) or static magnetic and electric fields (Penning trap). Quantum gates are applied by laser or microwave pulses. Transfer of quantum information between ions, enabling qubit entanglement, is realized through vibrational modes of the ion lattice.

There has been significant progress in the development of intermediate-scale quantum computers based on superconducting qubits with multiple devices being currently developed by academia and industry^30–32^. These qubits are made up of superconducting circuits with Josephson junctions to form an anharmonic quantum oscillator with discrete energy spectrum that can be addressed via microwave pulses^33^. Entangling gates are then realized through couplings between the circuits, for example via waveguide resonators. Over the past two decades, the coherence time of superconducting qubits has seen significant improvement^31^, and the artificial nature of these qubits has positioned superconducting quantum processors at the forefront in terms of sheer qubit count.

Linear optics were proposed as a possible platform for quantum computing already in 2001^34^. Recent developments focused on using single photon degrees of freedom for quantum computing in integrated photonic circuits^35^. Beam splitters, mirrors, phase shifters, wave plates and non-linear interactions in matter can be used to manipulate the quantum states of the photons, while single photon emitters and detectors provide and measure the photon states. Integrated photonic circuits try to scale this approach by miniaturizing these building blocks. Photons not only offer a digital quantum computing platform but also provide a pathway for measurement-based quantum computation, where quantum information processing is executed by performing measurements on entangled photon states^36–38^.

Besides superconducting qubits, other solid-state approaches are also explored as possible quantum computing platforms, e.g. nitrogen-vacancy centers in diamond^39^ as well as quantum dots^40–43^. Some of them are already used for analog quantum simulation^44,45^.

Note that beyond qubit plattforms also higher dimensional spaces, so-called qudits, are actively explored for various platforms^46–50^. Qudits extend the representation by employing *d*-level systems, potentially enabling more efficient quantum algorithms and enhanced information encoding capabilities.

Overall, superconducting circuits currently hold the record for the highest number of qubits and demonstrate fast clock speeds in quantum computing platforms. However, these systems often face limitations due to short coherence times. On the other hand, trapped ions offer better fidelities and longer coherence times, but controlling larger numbers of ions poses a significant challenge^51^. Photonic circuits, laser-cooled neutral atoms, and spin-based solid-state platforms, while displaying potential, still lag behind in terms scalability, fidelity and coherence times. Currently, every platform has a limited circuit depth that can be achieved before noise and gate imperfections result in the total decoherence of the computational state. These issues make it difficult to use all of the qubits for fault-tolerant quantum computation^52^ and for computations that are sensitive to these imperfections. Quantum error correction (QEC) is undeniably the pinnacle objective for quantum computing, particularly for DQS. However, achieving this goal on contemporary devices remains a distant endeavor.

### Digital quantum simulation on noisy quantum computers

As the hardware platforms continue to advance, an important question that arises is whether we can still benefit from DQS on current Noisy Intermediate-Scale Quantum (NISQ) devices^53^, notwithstanding their noise limitations. One of the first applications of DQS was using trapped ions, showcasing the digital method of quantum simulation^26^. While these results showed potential for scalable quantum simulation, the impact of noise, especially as the number of qubits and gates increases, remains a significant challenge to realize practical and accurate full-scale quantum simulations.

To overcome the noise limitations, a novel paradigm emerged in the NISQ era: the concept of variational quantum algorithms^54^. These are hybrid quantum-classical approaches which combine the ability of a classical computer to efficiently optimize scalar functions of multiple, real variables and of a quantum computer to represent states in high-dimensional Hilbert spaces and measure corresponding expectation values. The strength of variational quantum algorithms lies in their flexibility and tolerance to noise, as they involve shorter quantum circuits to handle complex problems. This comes at the cost of solving a high-dimensional optimization problem, which can exhibit the “barren plateau" phenomenon, where an exponentially vanishing gradient makes it difficult for classical optimizers to find directions for parameter updates, hindering algorithm convergence and efficacy^55,56^. For DQS phase estimation-based methods as well as Trotterized time evolution are more precise, if a fully error corrected quantum computer is available, but variational quantum algorithms can have significant advantages in the NISQ era.

In this perspective, we give a selective overview of the recent progress of DQS on NISQ devices. We separately discuss approaches that use variational quantum circuits and those that are based on non-variational methods. We continue with a discussion of the experimental and theoretical challenges, the impact of noise and decoherence and how error mitigation and hardware aware quantum algorithms can be used to tackle these challenges. We conclude with an outlook on the potential applications for DQS, going beyond the simulation of small spin systems in order to tackle realistic problems of quantum-many-body dynamics.

Note that this perspective serves as a qualitative overview on what can be done on actual quantum hardware, in what direction the algorithmic advances go and how the theoretical methods co-evolve with the architectural and experimental advances. Where possible we also try to directly compare the performance between experiments conducted on different platforms. However, this is often difficult due to varying initial conditions and different metrics being used.

## Non-variational results

In this section we discuss some of the recent key results that were achieved without using variational approaches. We discuss realizations of DQS on different hardware platforms with a focus on time evolution, simulation of topological systems as well as recent results on many-body localization and time crystals.

### Trotterized time evolution

The Trotterized approach to simulate the time-evolution of quantum many-body systems was the first proposed algorithm for quantum simulation with a provable quantum advantage^20^. The only assumption for this approach is that the Hamiltonian of the system *H* = ∑_*i*_*h*_*i*_ can be decomposed into polynomially many, local^20^ operators *h*_*i*_ that act only on a non-extensive number of qubits or which are Pauli string operators. This guarantees certain error bounds on the Trotter-Suzuki decomposition^57,58^ of the discretized time evolution operator according to $U(t)=\exp (-iHt)\, \approx \mathop{\prod }\nolimits_{n}^{N}{\prod }_{i}\exp (-i{H}_{i}t/N)$. Generalizations of the original Trotter approach are also known as product formulas.

Experiments using superconducting transmon qubits and trapped ions were performed on Ising, Heisenberg and other spin-1/2 models^26,59^, as they naturally map to qubits. While the general feasiblity was demonstrated in these studies, they also immediately identified the core problem of the Trotterized approach: its increasing circuit depth for larger time scales and the associated loss of fidelity due to the increased gate errors and decoherence. Fermionic models can be simulated by encoding via the Jordan-Wigner transformation^60^ in one spatial dimension or with tree-based encondings, such as the Braviy-Kitaev transformation^61^, in higher dimensions. A successful implementation was using superconducting quantum circuits^62^ with up to four fermionic modes. Also the dynamics of lattice gauge theories, specifically the Schwinger model, can be efficiently simulated using trapped ion quantum computers^63^. Here one of the main advantages of the trapped ion platform was used, as it can realize global entangling gates, in order to replace the gauge degrees of freedom in favor of long-range interactions in the qubit language.

**Fig. 1:**
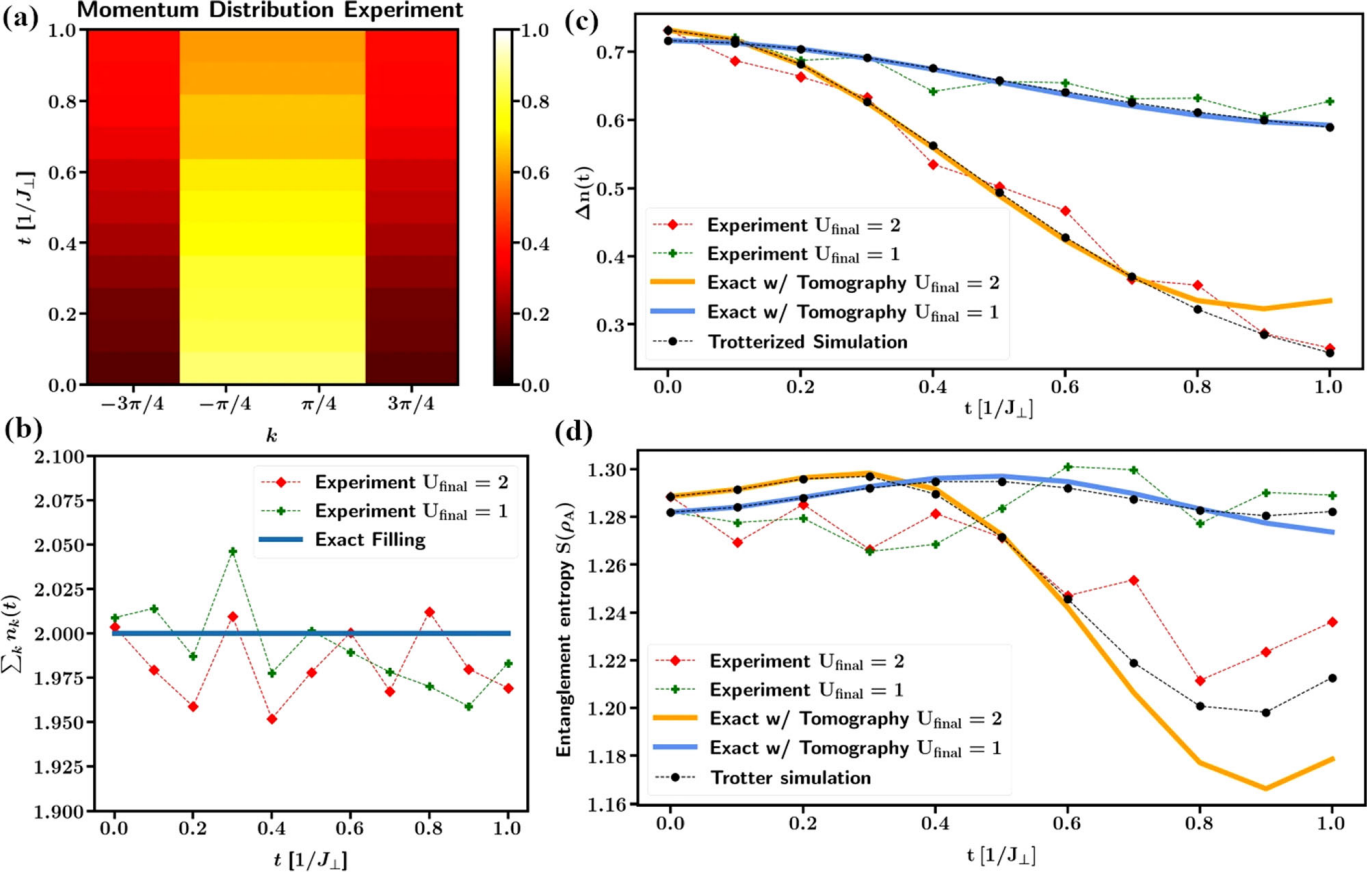
Digital quantum simulation of an interaction quench in a 1D fermion system with nearest neighbour interactions. Experimental data are obtained by combination of optimal state preparation, readout and symmetry error mitigation, zero noise extrapolation and full quantum state tomography after each time step on an IBM quantum computer. **a** Time and momentum dependence of the fermionic distribution function on a periodic four-site chain after an interaction quench to *U*_final_ = 2 at *t* = 0. **b** Time evolution of the filling factor. Comparison between the exact filling, the weak quench *U*_final_ = 1 and the strong quench *U*_final_ = 2. **c** Time evolution of the jump in the Fermi distribution. Comparison between the experimental results for weak and strong quenches and classical simulations. For the classical simulation, the state after initial preparation at *t* = 0 was obtained by full state tomography from the quantum computer. It was subsequently evolved by the Schroedinger equation, orange and blue line, and by Trotterization, black dashed line, on a classical computer. (d) Entanglement entropy of a bipartition of the system. The classical simulations were obtained with the same method as in (c). Figure adopted from^65^.

Recent work focused on optimizing the simulation using error mitigation approaches^64,65^ as well as using symmetries to reduce the computational effort^66^. It was also demonstrated that dynamical correlation functions, that can be measured in spectroscopic experiments, can be computed for spin systems^67^. In Fig. 1 we see an exemplary simulation of an interacting Fermi system on a chain using various error mitigation methods as well as optimized circuit compilation. This model is equivalent to the XXZ chain,

1 $$H=\mathop{\sum}\limits_{i\in {{\mathbb{Z}}}_{4}}\, {J}_{\perp }\left({S}_{i}^{x}\cdot {S}_{i+1}^{x}+{S}_{i}^{y}\cdot {S}_{i+1}^{y}\right)+U(t){S}_{i}^{z}\cdot {S}_{i+1}^{z},$$

by virtue of the Jordan-Wigner transformation. A quantum quench in the interaction *U* from 0 to *U*_final_ perturbs the Fermi gas and leads to a time-dependent reduction in the jump of the Fermi surface. Similar error mitigation methods allowed for the simulation of the Fermi Hubbard model with up to 16 qubits and the observation of spin charge separation on a superconducting quantum computer^68^.

The Trotterized approach is not only useful in order to calculate real-time dynamics, but it can also be used to compute thermal, excited and ground states using the quantum imaginary time evolution algorithm, as was first demonstrated on a superconducting quantum computer by Rigetti^69^. This approach has potential advantages over variational algorithms to prepare eigenstates, as it does not involve a multi-parameter optimization.

### Realizing topological systems

Quantum simulation of topological systems is not only a key problem for the theoretical description of various quantum materials but it is also directly at the interface to theoretical quantum information science as well as the experimental deployment of quantum error correction codes. A particular interesting class of codes, which are intrinsically local, are topological error-correction codes, such as the toric code and other surface codes^70,71^. In these codes the logical qubits are encoded in the topologically non-trivial surface, allowing for protection from decoherence through syndrome measurements and corresponding correction, which requires only physically local gates.

To identify topological states it is possible to make use of the bulk-boundary correspondence^72^, which relates topological bulk properties with robust edge states. By designing topologically protected quantum circuits it is possible to simulate topological systems and identify edge states, for example for one dimensional topological Floquet phases, as recently demonstrated on IBM and Rigetti quantum processors^73^.

**Fig. 2:**
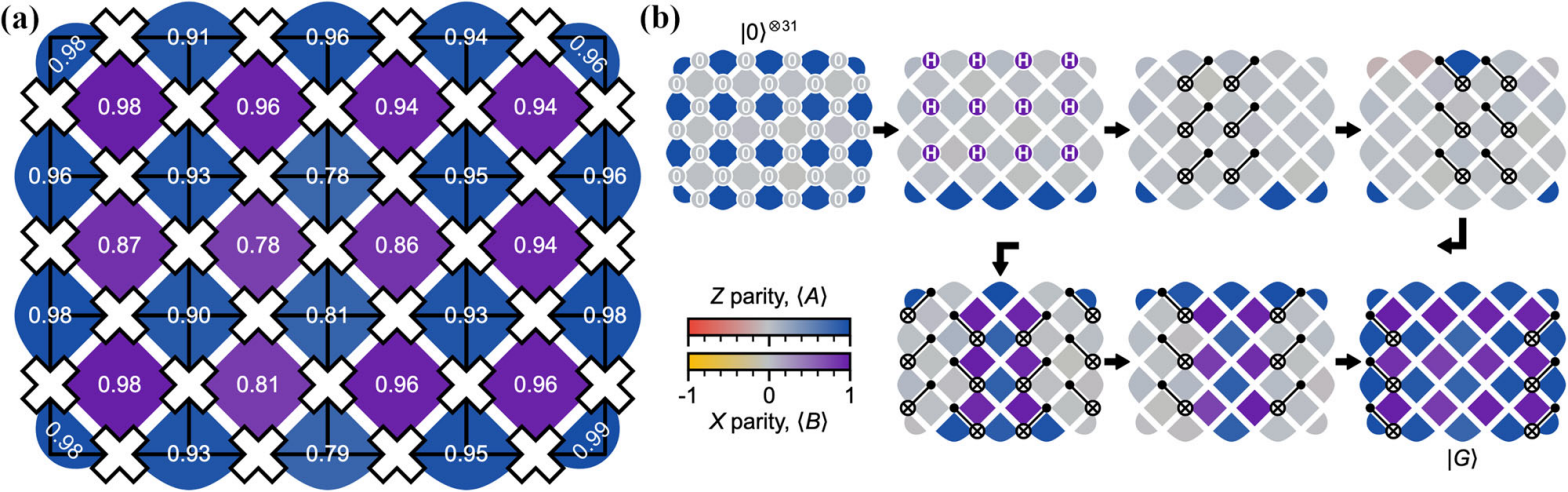
Toric code on a superconducting quantum processor. **a** Graphical representation of the star and plaquette parity measured after preparation of the toric code ground state. Qubits are represented as white crosses, solid black lines in the background show the lattice of the toric code. **b** Quantum circuit for the preparation of the toric code ground state on a 31 qubit processor. Figure adopted from^74^.

While this demonstrates a first step towards realizing topological states on quantum computers, for quantum error correction it is even more important to construct many-body topologically ordered systems with true long-range entanglement. Experiments for the toric code using Google’s superconducting Sycamore quantum processor were able to prepare the topologically ordered ground state to high accuracy and measure the entanglement entropy^74^. Figure 2 shows the parity of the star and plaquette operators after ground state preparation as well as the circuit employed to prepare the state. Also the braiding statistics of the anyonic excitations can be simulated, demonstrating a key property of topologically ordered systems^74,75^. Interestingly in a separate approach the toric code was also recently implemented in an analog fashion using a two-dimensional array of Rydberg atoms held in optical tweezers^18^, showing that both digital and analog approaches are converging towards artificially creating topologically ordered states.

In another work the phase transition to topologically protected phases was demonstrated using the IBM superconducting quantum computers^76^. Here the authors used a connection of the infinite size systems that can be iteratively generated using local quantum circuits and the fact that observables with finite support require only a finite size system to evaluate their value in the thermodynamic limit.

Another form of topological states that has recently been realized on trapped ion quantum computers^77^ and superconducting qubits^78^ are Floquet and quasiperiodically driven symmetry-protected topological phases. Especially the quasiperiodically driven system might prove to provide stable edge states robust against coherent errors.

### Many-body localization and time crystals

Many-body localized phases are protected by nonergodicity and the system fails to thermalize under its intrinsic dynamics. Here thermalization refers to the fact that in many-body systems any quantum states $\left\vert \psi \right\rangle$ with energy *E*_0_ will typically evolve after a certain time *t* to a state in which all few-body observables are well described by a thermal ensemble with a temperature determined by the energy of the initial state, allowing for a description of the system with statistical mechanics. This behavior breaks down in many-body localized phases due to disorder, which hinders thermalization similar to Anderson localization in non-interacting systems^79^. This interesting observation explains the hope of using many-body-localized states to protect quantum information even away from low temperature states in the presence of disorder.

Disorder tunable many-body localization has been realized with an analog trapped ion platform^80^ in a long-range transverse field Ising model with 10 qubits and later with up to 53 qubits^10^. A similar model was recently investigated using IBMs superconducting quantum computers^64^, where the qualitative behavior for up to 10 qubits was reproduced but without being able to reach the long-time limit. To limit the impact of the noise in near term devices it was proposed to measure the spectral functions of local operators, which retains signatures of localization even if a thermal bath destroys most other features of localization. The principal feasability of the approach was demonstrated for a 3 qubit system using a trapped ion quantum computer^81^.

Many-body localized phases are crucial in order to realize the famous time crystal in periodically driven quantum systems^82,83^, which is a subharmonic frequency response with coexisting long-range order which spontaneously breaks the discrete time translational invariance. As such, time crystals and their realizations are currently the focus of many research groups with advances realized through analog quantum simulation and computing devices. First experiments using NV-centers in diamond^84^ and trapped ions^85^ seem to confirm the theoretical predictions and are promising first steps to investigate such novel phases. However, detecting the true long-range nature of the spatiotemporal order is still out-of-reach for these analog experiments^86^.

Here digital quantum computers could provide an alternative route to realize and investigate time crystaline order experimentally^87^, allowing for Ising type disorder to be implemented and a wide array of initial states to be tested. Crucially digital quantum computers allow to discriminate between transient subharmonic responses and asymptotic time crystals. This idea was first realized on a superconducting quantum computer^88^ using a linear chain of up to 20 qubits. By varying the drive parameters the stability of the nonequilibrium phase was demonstrated and by a scaling analysis of the spectral properties the eigenstate order was confirmed. In another study a similar approach was successful in demonstrating a discrete time crystal using 57 qubits of IBM’s superconducting quantum computing platform^89^.

### Other non-variational results

Various other DQS approaches have been employed in recent years that could provide novel insight into many-body systems that are out of reach to simulate using state-of-the-art classical hardware.

Simulations using trapped ions and cold atoms demonstrated the ability of these platforms to simulate open quantum systems^90,91^. Recently, open quantum systems have also been addressed with new algorithms on superconducting quantum computers, allowing for the simulation of both Markovian and non-Markovian dynamics^92,93^. Systems with confinement were simulated with IBM quantum computers^94^. Out-of-time-ordered correlators (OTOCs) are a measure of the degree of quantum chaos in a system. They are used to determine how quickly information becomes scrambled within a quantum system. OTOCs have been measured on trapped ion quantum computers^95,96^, on an NMR quantum simulator^97^ as well as on superconducting platforms^98,99^ including a measurement on a seven qutrit processor^100^.

## Variational results

In this section we discuss key results in DQS using variational quantum algorithms in a classical-quantum feedback loop. As such they operate with two main components: a quantum computer, which encodes the state of the system via a quantum circuit, and a classical computer, which optimizes the parameters of the quantum circuit to minimize a cost function. To evaluate the cost function measurements on the quantum state are performed and sent to the classical computer, which computes the cost function and optionally its derivative. In an iterative scheme the classical optimizer then proposes updated parameters of the circuit, which are sent to the quantum computer in order to find the optimal solution to the problem.

We will give a short overview on the development of variational quantum algorithms and then focus on ground and excited state properties as well as quantum many-body dynamics computed by variational approaches. Other variational results relevant for DQS are also discussed.

### Overview on variational quantum algorithms

Variational quantum algorithms have been proposed for machine learning tasks, for combinatorial optimization problems, as numerical solvers for factorization, matrix decompositions and differential equations, as well as for quantum compilation^54,101^. The variational approach is especially useful for the NISQ era, as it allows a tradeoff between variational accuracy and loss of coherence due to gate imperfections and noise, by varying the depth of the variational circuit.

In the context of DQS variational quantum algorithms can be compared to classical variational methods, such as tensor networks^102^ and variational Monte Carlo^103^. The main advantage of quantum states prepared by parameterized quantum circuits is an enhanced expressivity when compared to low-rank tensor states that can be classically computed^104,105^. Expressivity can be measured by investigating how much entanglement entropy $S({\rho }_{A})=-{{{{{{{\rm{Tr}}}}}}}}({\rho }_{A}\ln {\rho }_{A})=-{{{{{{{\rm{Tr}}}}}}}}({\rho }_{B}\ln {\rho }_{B})=S({\rho }_{B}),$ of a bipartite system can be efficiently captured by the variational wave function. As such, parameterized quantum circuits could prove to be a useful tool in order to describe complex many-body ground and excited states as well as time evolved states in non-equilibrium systems. Thus a significant research effort is put into evaluating the performance of these approaches in comparison to classical methods, as well as the effect of noise and gate imperfections, which can quickly lead to a breakdown of the potential quantum advantage.

Another field of research concerns the optimization of the variational parameters. As the number of variational parameters increases, performance of classical optimizers can decrease significantly. This can be attributed to multiple factors, such as the interaction between noise and large number of parameters, as well as the formation of Barren Plateaus^56^. New ways to optimize such non-linear functions evaluated on quantum computers are currently explored^106^.

### Ground and excited state properties

The first variational algorithm that was employed on a photonic quantum computer is the variational quantum eigensolver (VQE)^107^. The basic idea of the VQE approach is to approximate the ground state of a quantum system via a parameterized quantum circuit. The target function is therefore the energy of the variational state $L(\theta )=\left\langle 0\right\vert {U}^{{{{\dagger}}} }(\theta )HU(\theta )\left\vert 0\right\rangle$, where *U*(*θ*) is the unitary corresponding to the parameterized quantum circuit with variational parameters *θ*. In the first application of the VQE algorithm, a 2-qubit photonic processor was used with a single CNOT gate and adjustable phase shifters to compute the bond dissociation curve of the He-H^+^ molecule.

In comparison to QPE, which estimates energy eigenvalues through coherent time evolution of the Hamiltonian^5^, VQE has significantly reduced requirements for coherent gate execution. In a direct comparison between VQE and QPE to calculate the energy curves of molecular hydrogen, VQE demonstrated superior performance and a robustness towards errors in the gate implementations on a superconducting quantum processor^108^. This can be traced back to the deeper and more complex circuit of the QPE algorithm, requiring controlled Trotterized time evolution, as well as the ability of VQE to adjust for gate imperfections by further optimization of the variational parameters.

**Fig. 3:**
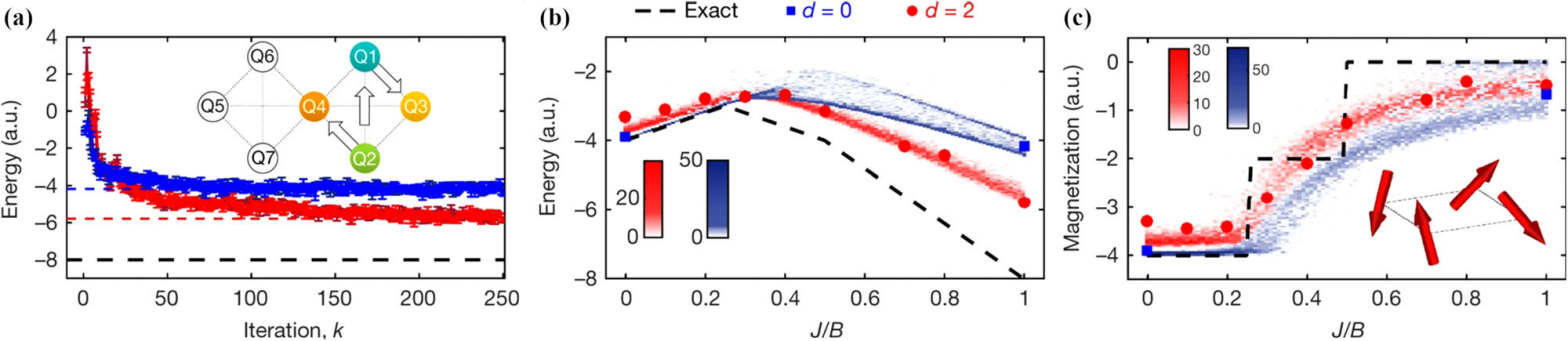
Variational Quantum Eigensolver applied to a four qubit Heisenberg model on a superconducting quantum computer. **a** Energy optimization using a hardware efficient Ansatz of depth 0 (blue) and depth 2 (red), with dashed lines indicating the final energy estimate for *J*/*B* = 1. Plotted along with the exact energy (dashed black line). The inset highlights the qubits used for the experiment along with the entangling gates (arrows) (b), (c) Experimental results (blue and red data points) plotted along with exact values (black dashed lines) and density plots of 100 numerical outcomes, for energy (b) and magnetization (c), for a range of values of *J*/*B*. Figure adopted from^110^.

Due to the success of the VQE approach new questions arise in order to scale its applicability beyond simple two qubit examples. One of the key questions concerns the ansatz of the parameterized quantum circuit in order to represent the variational ground state. In this context, parallels to classical machine learning^109^ emerge, as the optimal ansatz should both represent the target state efficiently while at the same time beeing as shallow as possible. One particular appealing class of ansatz schemes are hardware-efficient quantum circuits. These circuits are designed having the hardware capabilities in mind; for example by limiting the use of noisy entangling gates, by using hardware intrinsic gates to design the ansatz and by avoiding costly swap gates in quantum processors with limited connectivity. Using hardware-efficient quantum circuits, small molecules and a four-qubit Heisenberg model in a magnetic field *B*

2 $$H=J\mathop{\sum}\limits_{i,j}{\overrightarrow{S}}_{\!\!i}\cdot {\overrightarrow{S}}_{\!\!j}+B\mathop{\sum}\limits_{i}{S}_{i}^{z}$$

were simulated on an IBM superconducting quantum processors^110^. Figure 3 shows the results of the variational optimization for energy and magnetization in the system depending on *J*/*B*. One of the largest ground state VQE calculations has been performed to simulate a quadratic Hamiltonian evolution with up to 12 qubits, computing the binding energy of hydrogen chains and the isomerization mechanism of diazene^111^. The experimental results demonstrate the improvement of the variational approach with circuit depth and the capability of the quantum circuit to yield qualitatively correct results while still being subject to noise.

Replacing QPE with VQE improves on the required depth of the quantum circuit but increases the number of measurements due to the optimization procedure. This makes VQE less attractive for platforms which have a low clock frequency, e.g. trapped ion platforms. Here progress was made using a trapped ion platform with up to 4 qubits in combination with VQE to compute the ground state properties of small molecules^112,113^ using a minimal unitary coupled-cluster ansatz state. In another work the Schwinger model was simulated with up to 20 qubits^114^ using a hybrid approach combining an analog quantum simulator with a classical computer.

Beyond ground states it was proposed to use the variational approach also to compute excited states on quantum computers^115,116^. The most straight forward approach to compute excited states is to implement a Gram-Schmidt orthogonalisation procedure, by modifying the target function,

3 $$L(\theta )=\left\langle 0\right\vert {U}^{{{{\dagger}}} }(\theta )HU(\theta )\left\vert 0\right\rangle+\lambda \mathop{\sum}\limits_{i}\left\langle 0\right\vert {U}^{{{{\dagger}}} }({\phi }_{i})U(\theta )\left\vert 0\right\rangle$$

where *λ* is a Lagrange factor that penalizes overlap of the trial state $U(\theta )\left\vert 0\right\rangle$ with previously found solutions $U({\phi }_{i})\left\vert 0\right\rangle$, such as the ground state. This approach was implemented to calculate excited states of a non-Abelian gauge theory coupled to matter on an IBM quantum computer using up to 6 qubits^117^, aiming for the simulation of quantum chromodynamics. The required overlap was computed using forward-backward propagation with ${U}^{{{{\dagger}}} }({\theta }_{{{{{{{{\rm{ex}}}}}}}}})U({\theta }_{{{{{{{{\rm{gs}}}}}}}}})\left\vert 0\right\rangle$ and comparison of the measurement result with the initial state $\left\vert 0\right\rangle$.

Another interesting application has been proposed through variationally diagonalizing mixed quantum states^118^, which is useful to spectroscopically identify topological properties of quantum phases. It was demonstrated for a simple one-qubit example on Rigetti’s quantum computer.

### Quantum many-body dynamics

While the Trotterized time evolution is one of the first non-variational algorithms that was proposed and experimentally realized in DQS, variational approaches first focused on approximating eigenstates instead of time evolution. By now there are several proposals^119–122^ solving an equation of motion or optimizing variational parameters following the McLachlan time-dependent variational principle to implement the time-dependent Schrödinger equation for parameterized quantum circuits. Another proposal is to variationally compile the Trotterized time evolution into a diagonal form to overcome the decoherence limit of the quantum computer. The theoretical reason why also time dependent wave functions should be well approximated by parameterized quantum circuits is that local Hamiltonians stay within an exponentially small Hilbert space of the system^123^, although not necessarily in a space that is efficiently simulatable classically.

One of the first methods for variational time evolution that was implemented is based on variational fast forwarding (VFF) the Trotterized time evolution^124^. VFF approximates a diagonalization of the short-time simulation to enable longer-time simulations with a fixed number of gates by using the quantum-assisted quantum compiling algorithm^125^. The approach was implemented using a two qubit circuit on Rigetti’s quantum computer for a randomized quantum circuit. A modified fixed state VFF^126^ diagonalizes the energy subspace spanned by the initial state resulting in improved requirements on circuit depth. It was successfully implemented on IBM and Rigetti quantum computers simulating a two-qubit XY spin model.

A hybrid algorithm that utilizes a qubit representation of the density matrix instead of a variational wave function has been developed^127^ to implement quantum time evolution. The density matrix is time evolved for each element and its matrix elements are computed on a classical computer using density matrix quantum Monte Carlo. The performance of this approach is highly dependent on the sparsity of the density matrix. The algorithm has been implemented on Rigetti quantum computers for a single qubit.

Another approach that does not require a quantum-classical feedback loop is quantum assisted simulation (QAS)^128^. It is using so called K-momentum states to derive an equation of motion for the variational parameters of the wave function. The states are initially prepared on the quantum computer and their overlap and energy expectation value are measured and used as an input for the equation of motion. The principal feasibility of the approach was tested on a two qubit circuit using the IBM quantum computers. The main disadvantage of the approach is that it is unclear how well the states approximate the long-time evolution and that a matrix inversion on noisy measurement results is necessary, which is often unstable.

Recently a first implementation of the variational time evolution based on optimization was implemented on IBM quantum computers simulating the Heisenberg model with 3 qubits^129^. The results suggest an improvement over the pure Trotterized approach but simulations for larger systems also demonstrate that the required circuit depth scales linearly with the entanglement induced due to the time evolution.

## Challenges and methods in digital quantum simulation

To continue the pursuit of quantum speed-ups in DQS, it is vital to address current challenges and limitations in the field. This includes advancing quantum hardware technology, verifying performance guarantees and developing improved algorithms. The primary source for errors in current day devices is mainly due to decoherence and gate imperfections. Although the hardware performance has significantly increased for various platforms in the recent years^25,31^, it remains an open question if and which technology will first achieve the necessary requirements for fault tolerant quantum computation^130^. Before the age of fault tolerance, quantum advantage, i.e. the ability to simulate quantum systems and solve complex problems that are intractable for classical computers, might be achievable with NISQ devices if hardware-software co-development can be leveraged, which vertically integrates the design of quantum computers with their potential applications^131^.

### Challenge: Noise and decoherence

When multiple qubits are connected and operated together, new challenges arise such as the need to address specific qubits^25^ and manage cross-talk errors^31,132^. These issues can result in slower and less accurate gates, which requires proper evaluation of the capabilities of a quantum computer, depending on the number and quality of the qubits, as well as the use of optimal control theory to tackle fidelity issues^133^.

One area of particular interest is the simulatable system size, taking into account that current state-of-the-art platforms can have ∝ 100 qubits available for use^134^. From a DQS standpoint, this number is actually sufficient for a wide range of applications. For example, simulating a 10x10 Fermi-Hubbard model would require 200 qubits and the results would be highly interesting for various research questions in many-body systems^135^. However, current hardware does not allow using all the qubits on the device, even with a very shallow quantum circuit, without succumbing to the noise and decoherence wall. This is reflected in various benchmarks, such as the quantum volume^136–138^, which measures the effective number of qubits that can be used on a given device based on a randomized benchmark protocol. Across various quantum computing platforms the maximum achievable quantum volume translates into at most 15 effectively usable qubits^139^, far from what is required for full error correction but also much less than what is needed for a general quantum advantage in DQS. This makes it crucial to focus on improving the operational fidelities of single physical qubits and coherence times of entangled states created during the computation, addressing the limited system size issue for DQS while also improving the general computation capabilities of NISQ devices.

### Pre- and postprocessing procedures

Quantum error mitigation describes various methods that have been proposed to help increasing the applicability of NISQ devices. These methods, while independent of the actual application, are important for DQS to increase the accuracy of observables and to improve the optimization procedure in variational methods by lowering the error of the target function. Here, we discuss some of the most important techniques employed so far, in particular readout error mitigation, zero noise extrapolation and optimized circuit compilation. Depending on the hardware, other mitigation techniques can be more important that those discussed here, but the principal theme of these approaches remains the same.

Readout error mitigation is a technique used to correct for errors that occur during the measuring process. By performing multiple measurements of the same qubit state it is possible to use a statistical method to suppress readout errors. This can be done by measuring the qubit in multiple bases and using a maximum likelihood estimation algorithm to infer the true state. One of first readout error mitigation schemes was applied to two superconducting transmon qubits^140^. The method was extended using quantum detector tomography an tested on IBM and Rigetti quantum computers^141^. In its pure form readout error mitigation requires calibration measurements on all possible quantum states, leading to an exponential increase in required measurements. However, if the readout errors are only partially correlated, e.g. due to the local structure of the qubits, tensored readout error mitigation overcomes this issue^142^.

To also address noise during the execution of the quantum circuit zero noise extrapolation has been developed in the context of many-body simulations^119^ and later expanded to other quantum circuits^143^. It is based on the idea of Richardson’s deferred approach to the limit by systematically repeating quantum computations at varying noise levels and then extrapolating the results to the hypothetical zero-noise limit. Varying noise levels are achieved by systematically stretching the physical pulses, increasing their pulse duration while reducing their amplitude. On IBM’s superconducting quantum computers this approach was applied to the single as well as to the two-qubit gates, by rescaling the duration of the microwave pulses^144^.

Quantum circuit compilation is the process of translating a high-level quantum algorithm into a corresponding low-level quantum circuit that can be executed on a real device^145^. This process is necessary due to the limitations of current quantum hardware, such as qubit dependent levels of noise and limited connectivity between qubits. The goal of quantum circuit compilation is to find the most efficient and robust implementation of a quantum algorithm that can be executed on a given hardware platform. As such quantum circuit compilation is highly hardware dependent and various approaches are currently explored in the literature^146,147^. Optimized state preparation with a minimal number of entangling CNOT gates can be realized based on isometry decompositions^148^ and were used for the quantum simulation of quenched fermionic systems on IBM quantum computers^65^.

Combining these error mitigation methods leads to a synergistic effect that could be crucial to reach a quantum advantage over classical computers. Recently, a combination of error mitigation techniques was used to compute the quench dynamics in an Ising model^149^ using 26 qubits of an IBM quantum computer. Here dynamic decoupling^150^, Pauli twirling^151^, native gate decomposition, readout error mitigation and zero noise extrapolation demonstrated the practical scaling of these methods to larger system sizes and their capability to significantly enhance the performance of the hardware.

### Hardware aware quantum algorithm development

With the availability of NISQ devices also the development of quantum algorithms has adapted to the available hardware features and drawbacks. The central challenge is utilizing noisy quantum computers to gain a quantum advantage. This requires a plan that takes into consideration the restricted number of qubits, the qubits’ connection limitations, and the errors that affect coherence, state preparation, readout and thereby circuit depth. At the same time it means developing novel algorithms that use existing features, such as decoherence free subspaces^152^ and multi-level qudit platforms^47^.

For DQS, variational algorithms promise flexible scalability of circuit depth at the cost of limited knowledge about the variational fidelity and additional measurement costs due to the non-linear optimization^54^. Beyond the previously discussed VQE approach, other methods have been proposed to prepare ground states, e.g. for fermionic Hamiltonians using antisymmetrization of initial states, QPE and qubitzation^153,154^. Hardware-efficient variational time evolution using optimization and time dependent variational principle has been proposed and classically simulated for quantum Ising chains^155^. Algorithms for computing the Green function based on variational time evolution and on a Lehmann representation of the spectral function have been developed^156^. A similar approach for magnon spectra has been proposed and tested on IBM quantum computers^157^. Quantum computation of Floquet spectra in periodically driven many-body systems can be enhanced using multi-level qudit quantum computing platforms^158^. These are just a few examples of the many different types of quantum algorithms that have been developed and proposed for DQS. For an overview of other quantum algorithms we refer to other reviews^54,159,160^.

## Outlook and applications

The advances in digital quantum simulation have been comprehensively reviewed in the preceding sections. Here, I wish to present my own viewpoint on their wider implications.

Digital quantum simulation is a rapidly growing field that has the potential to have a significant impact on the study of quantum many-body systems. The ability to simulate intermediate scale spin systems using near term quantum devices offers the possibility to discover new phenomena and improve our understanding of previously observed phenomena. However, in order to fully realize the capabilities of DQS, it will be important to focus on simulating more realistic and complex problems.

As we think about the most promising problems to be tackled with DQS on near-term devices, I think it is prudent to emphasize that problems of qualitative nature have a more promising outlook than those aiming purely for a quantitative improvement in accuracy. The examples discussed in the results section exhibit significant strides towards a quantum advantage, yet they remain largely confined to small system sizes due to the inherent limitations of noise and gate errors.

Recently advanced error mitigation techniques^161^ were used to push the boundaries of system sizes into the regime of 100 qubits to simulate the time evolution of a 2D transverse field Ising model. However, these techniques encounter difficulties when the circuit depth reaches and goes beyond the device’s decoherence time, resulting in an exacerbation of existing issues rather than their resolution. Therefore, a pursuit of improved quantitative accuracy, i.e. by increasing circuit width and depth, could inadvertently magnify these challenges. To provide a concrete example consider reaction rates in quantum chemistry, which depend exponentially on the accuracy of the energy in the simulation leading to a significant effect in the predicted reaction. This sets a natural limit on the required accuracy for such calculations^162^, which is unrealistic to achieve for NISQ devices while they also have to compete with state-of-the-art numerical approaches. On the algorithmic side its even unclear, if one of the main tasks in quantum chemistry, i.e. ground state energy estimation, can achieve an exponential quantum advantage at all^163^.

In my opinion, this highlights the necessity of shifting our focus towards problems that involve fundamental principles and qualitative understanding. Such problems beckon us with questions that are foundational in character, often concerning the very nature of quantum states, their stability, phase transitions, and symmetries.

A better understanding of the topologically ordered states in the fractional quantum hall effect^164,165^, the nature of the superconducting glue in unconventional superconductors^166,167^, the ability to induce quantum phases “on demand" via ultrafast excitations^3^, and the identification of interesting quantum materials such as topological insulators, superconductors, and spin liquids^168^ are fundamental problems that in many cases are hard to describe with classical methods, especially if the corresponding quantum states are highly entangled.

While powerful classical methods exist for approximating low-dimensional ground states, they often reach their limits in systems that require large cluster sizes for accurate representation, posing significant challenges to the scalability and precision of numerical methods. Take, for example, the debate concerning the ground state competition between charge and spin ordered states versus superconductivity in the 2D square lattice Hubbard model pertinent to the cuprates^169,170^. The true ground state, in particular in the underdoped regime, is still an active field of research^171^, despite the simplicity of the model. The formation of wavelength *λ* = 8 stripe order seems to coexist with d-wave superconductivity, but it is unclear if an even longer wavelength could compete with this state or another competing ground state forms. Such calculations would require cluster sizes that are out of reach for current but likely also next-generation numerical approaches. Here, DQS on larger clusters could offer qualitative insight into potential novel low energy states in the near term, even though the energy measurements may initially have large error bars, making it uncertain if the DQS solution truly represents the ground state. However, two key aspects are noteworthy: first, hardware improvements will directly reduce these error bars over time, and second, new insights into the low energy sector, including meta-stable states, are of significant interest. Such states, potentially stabilized by minor system perturbations, could provide crucial information for understanding and manipulating these systems.

As another example, non-equilibrium dynamics in many-body systems presents persistent challenges, especially when addressing phenomena such as light-induced superconductivity^172–174^, collective excitations^175–178^ and combinations of the two^179^. Classical computational methodologies often face difficulties in capturing these dynamics due to their complexity and the pivotal role of high-energy states, which are frequently omitted in many low-energy approximations. This limitation can restrict our comprehensive understanding and might bypass essential mechanisms inherent to the phenomenon. DQS, while not achieving the quantitative accuracy of some classical methods, has the potential to provide qualitative insights that could enhance our understanding of many-body non-equilibrium dynamics.

Beyond the pure unitary case, monitored quantum dynamics^180^ has recently attracted attention as a possible application for near term devices. It lies at the intersection of quantum information science and condensed matter physics and investigates quantum circuits, which are constructed from local unitary gates and measurements. Complex questions that arise in this context concern thermalization, entanglement, and quantum chaos. Notably, their dynamics can both echo traditional quantum behaviors and introduce novel phenomena absent in conventional settings. One such novel behavior is the entanglement phase transitions in quantum systems when they are under external observation^181^, with first successful experiments^182^ performed on trapped ion platforms. Many qualitative questions remain unanswered, particularly in the two and three-dimensional case and in open quantum systems, as monitored quantum dynamics is leading to a rapid proliferation of quantum trajectories. This intricacy can potentially offer a quantum advantage sooner than pure unitary systems, as quantum devices can inherently handle such dynamics more efficiently than classical computers.

With the further development of digital quantum computers, it will be possible to address a wide range of open questions in quantum matter. Nevertheless, the problems that we will be able to tackle will grow iteratively and it is essential to acknowledge that each leap forward hinges not just on advancements in quantum hardware but also on refining the entire hardware-software ecosystem.

As of now, a definitive quantum advantage in any DQS application remains unproven. However, for researchers who investigate areas with qualitative questions surrounding many-body systems, it may be prudent to forge collaborations with the quantum computing community. Such partnerships can spur the identification of new use cases and the development of near-term algorithms, not only addressing specific challenges but also paving the way for the much-anticipated demonstration of the first genuine quantum advantage on digital quantum devices.

**Fig. 4:**
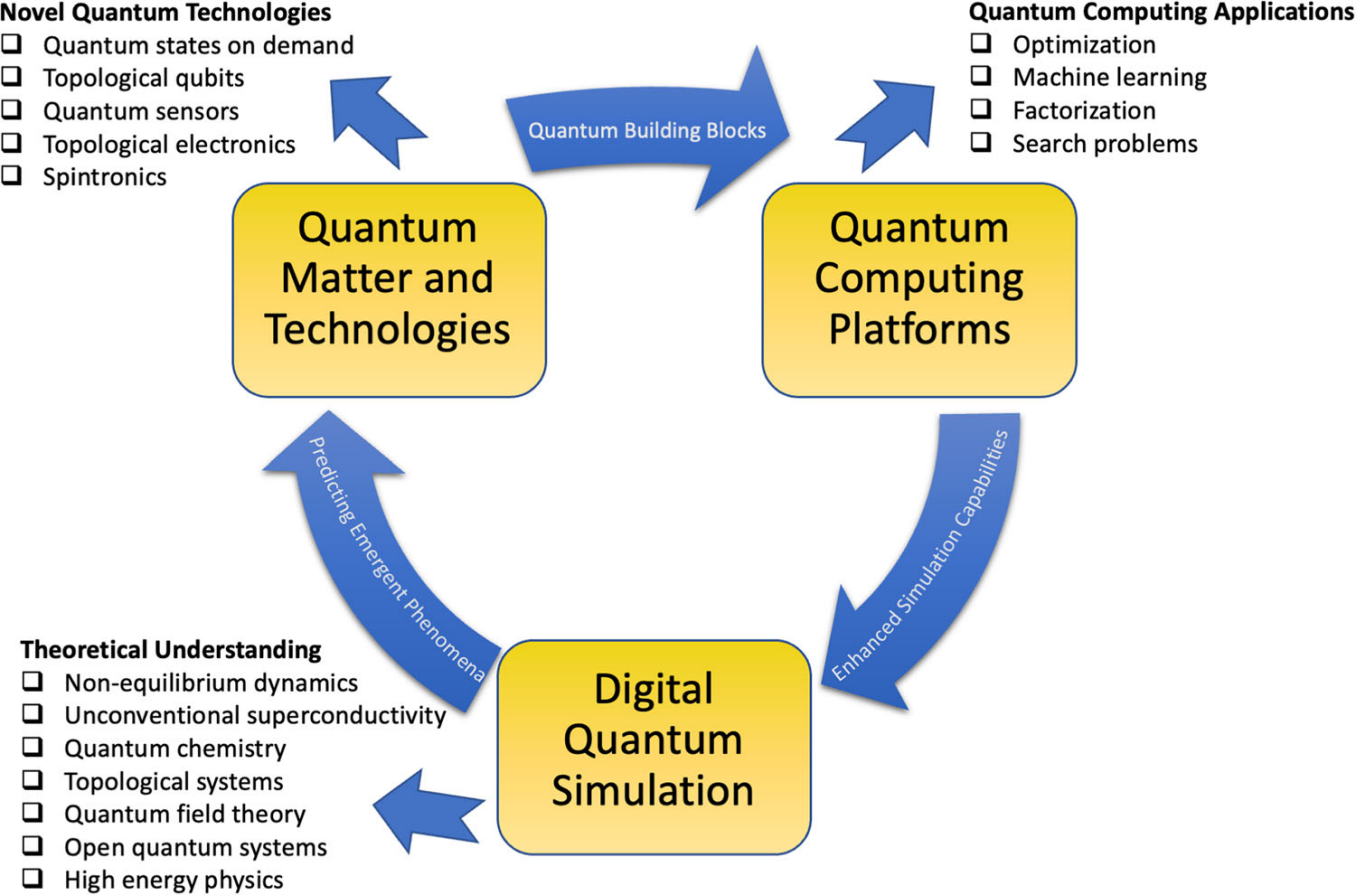
Quantum Technology Circle. Relation between the fields Digital Quantum Simulation, Quantum Matter and Technology and Quantum Computing Platform development, which could potentially foster a Quantum Moore’s Law. Current state of the art quantum platforms are the results of years of research on complex many-body systems and quantum matter. Quantum computing platforms enable digital quantum simulation with the potential to predict emerging states, which in turn support the development of novel quantum building blocks that can potential serve quantum computing platforms. At each point of the circle there are spin-off applications and technologies which can be useful in other fields, such as the understanding of non-equilibrium phenomena.

In the long-term, the advancements in DQS could potentially enable a full quantum technology circle, see Fig. 4, where quantum computing is used to enhance the simulation capabilities in order to understand and develop novel quantum technology, which in turn further enhances the quantum processing power. As of now the potential power of this circle is only partially tapped. While current feedback loops in quantum computing development, like those implemented by companies such as IBM, effectively integrate hardware and benchmark improvements, these processes have not yet systematically incorporated DQS, leaving untapped potential for a more holistic integration. While there are existing proposals focusing on a full quantum computing stack^131^, marrying hardware development with applications, I believe that there is a need to further broaden this perspective by creating a harmonized stack that seamlessly integrates quantum technology, quantum computing hardware, and DQS application development. In such an ecosystem, quantum phases on-demand and the exploration of exotic quantum matter will be foundational pillars for the underlying quantum hardware. By fostering these interconnections, we might be able to realize a quantum version of Moore’s Law, marked by ongoing hardware self-improvements and an enriched understanding of quantum many-body physics.
